# Development of a novel self-healing Zn(II)-metallohydrogel with wide bandgap semiconducting properties for non-volatile memory device application

**DOI:** 10.1038/s41598-024-61870-1

**Published:** 2024-06-07

**Authors:** Arpita Roy, Subhendu Dhibar, Kripasindhu Karmakar, Subham Bhattacharjee, Bidyut Saha, Soumya Jyoti Ray

**Affiliations:** 1 Department of Physics, Indian Institute of Technology Patna, Patna, Bihar 801103 India; 2 Colloid Chemistry Laboratory, Department of Chemistry, The University of Burdwan, Golapbag, Burdwan, West Bengal 713104 India; 3 Department of Chemistry, Kazi Nazrul University, Asansol, West Bengal 713303 India

**Keywords:** Zn(II)-metallohydrogel, LMWG, Self-healing, Injectable property, Microstructure, Semiconducting device, Resistive switching, Neuromorphic computing, Gels and hydrogels, Electronic and spintronic devices, Supramolecular polymers, Ligands

## Abstract

A rapid and effective strategy has been devised for the swift development of a Zn(II)-ion-based supramolecular metallohydrogel, termed Zn@PEH, using pentaethylenehexamine as a low molecular weight gelator. This process occurs in an aqueous medium at room temperature and atmospheric pressure. The mechanical strength of the synthesized Zn@PEH metallohydrogel has been assessed through rheological analysis, considering angular frequency and oscillator stress dependencies. Notably, the Zn@PEH metallohydrogel exhibits exceptional self-healing abilities and can bear substantial loads, which have been characterized through thixotropic analysis. Additionally, this metallohydrogel displays injectable properties. The structural arrangement resembling pebbles within the hierarchical network of the supramolecular Zn@PEH metallohydrogel has been explored using FESEM and TEM measurements. EDX elemental mapping has confirmed the primary chemical constituents of the metallohydrogel. The formation mechanism of the metallohydrogel has been analyzed via FT-IR spectroscopy. Furthermore, zinc(II) metallohydrogel (Zn@PEH)-based Schottky diode structure has been fabricated in a lateral metal–semiconductor-metal configuration and  it’s charge transport behavior has also been studied. Notably, the zinc(II) metallohydrogel-based resistive random access memory (RRAM) device (Zn@PEH) demonstrates bipolar resistive switching behavior at room temperature. This RRAM device showcases remarkable switching endurance over 1000 consecutive cycles and a high ON/OFF ratio of approximately 270. Further, 2 × 2 crossbar array of the RRAM devices were designed to demonstrate OR and NOT logic circuit operations, which can be extended for performing higher order computing operations. These structures hold promise for applications in non-volatile memory design, neuromorphic and in-memory computing, flexible electronics, and optoelectronic devices due to their straightforward fabrication process, robust resistive switching behavior, and overall system stability.

## Introduction

Nature provides attractive functional materials with elegant architecture through the integration of simple interactions that promote diverse applications in human life. Among them hydrogels is one of the most attractive soft-scaffolds with three-dimensional architecture.^1^ Supramolecular hydrogels are composed of low molecular weight gelator (LMWGs) ligands, with molecular masses < 3000, that immobilize the water solvent through various non-covalent interactions to form 3D networks^1^. Many low molecular weight gelators such as ethanolamines^2–4^, oxalates^5–7^, terpyridines^8,9^, fatty acid^10^, amino acid^11^ etc. have been reported for rapid gelation. LMWGs are assembled into gel networks through various non-covalent interactions^12^ such as hydrophobic-hydrophobic interactions, hydrogen bonds, metal coordination, van der Waals interactions, and π···π interactions, which allow their use in material science with different external stimuli like mechanical force, temperature, pH, and electric field etc^13,14^ Due to the tunable physical and chemical properties with 3D atmosphere, hydrogels have extensive applications in various biomedical fields such as tissue engineering, delivery vehicles for drugs, cells or proteins, contact lens, wound dressing, and artificial muscle etc^15–22^. However, in recent time hydrogels have been found to have several applications in bioelectronics^23^, optoelectronics^24–26^, energy devices^27,28^, semiconducting devices^29^, catalysis^30^, dye-removal^31^, memory^32^ etc.

Self-healing hydrogels are an important class of hydrogels that have the ability to repair its broken network without external stimulation. These “smart” hydrogels sustain and rejoin their network and mechanical properties even after being dented^33^. The literature is full of numerous reports about hydrogels but few of them show self-healing properties. In recent time, self-healing hydrogel have important outline in material science for their enormous application, especially in biomedical applications^33^. However many researchers have shown practical application of self-healing hydrogel in electronic devices ^34^ and electro-optics/photonics^35,36^. Metallohydrogels have become a trending topic in supramolecular chemistry, where metal-ions play a crucial role through metal–ligand coordination via diverse non-covalent interactions to complete the supramolecular gelation process. Supramolecular metallohydrogels have been used in various applications such as sensing^37–39^, catalytic property^40–42^, redox ^43^, magnetic^44,45^ etc.

On the other sides, Resistive Random Access Memory (RRAM), also known as ReRAM or memristor-based memory, is an emerging non-volatile memory technology that has garnered significant attention for its promising characteristics. RRAM stores information by utilizing the resistive switching effect in a metal–insulator-metal (MIM) structure, where the resistance state can be changed reversibly between high and low resistance states under the application of an electric field. One of the primary advantages of RRAM is its non-volatile nature which means it retains stored information even when power is turned off. This characteristic is essential for data storage applications and distinguishes RRAM from volatile memories like DRAM, SRAM. RRAM, exhibiting fast switching speeds and low power consumption during write and read operations. It is also suitable for energy-conscious applications, such as in battery-powered devices and IoT (Internet of Things) systems. These types of devices typically demonstrate long data retention times. The stability of stored data over extended periods is crucial for various applications, including archival storage and memory. RRAM technology offers scalability down to smaller feature sizes. This scalability is vital for achieving higher memory density to develop memory devices with larger capacities in smaller form factors. The fabrication process for RRAM devices is relatively easy compared to some other emerging memory technologies. This simplicity can contribute to cost-effectiveness in manufacturing. The analog switching behavior of RRAM devices has led to interest in their application in neuromorphic computing^46,47^. It has ability to mimic synapse-like behavior^64^ which makes it a potential candidate for implementing efficient and power-aware neuromorphic circuits in the field of artificial intelligence. It is being explored as a candidate for storage-class memory, a type of memory that bridges the gap between traditional volatile and non-volatile memories. In addition to conventional memory applications, RRAM's compatibility with flexible substrates and transparent conductive materials makes it suitable for integration into flexible and transparent electronic devices, including wearable electronics and displays.

However, RRAM holds great promise as a next-generation non-volatile memory technology with advantages such as non-volatility^54^, high-speed operation, low power consumption^55^, long retention time^56–59^, scalability, good manufacturability and simplicity in fabrication. It has wide range of applications, including non-volatile memory, embedded systems, artificial intelligence, flexible electronics, and neuromorphic computing which plays an important role in the future landscape of memory technologies. The switching process can be explained via several physical mechanisms, such as charge carrier entrapment and vacancy migration^67,68^, electrochemical migration etc. In our recent works, the resistive switching behaviour of oxide-based RRAM devices^46–51,53^ has been demonstrated. However, metallohydrogels can be useful candidates for the such memory design due to their semiconducting nature and ability to integrate in flexible electronics which can be useful for optical detection, sensing, and memory.

Through this work, we have demonstrated an eco-friendly route to develop a rapid supramolecular metallohydrogel of Zinc(II)-ion and pentaethylenehexamine in room temperature. Direct mixing of the low molecular weight gelator pentaethylenehexamine and aqueous solution of zinc nitrate hexahydrate generated stable Zn(II)-metallohydrogel (i.e. Zn@PEH) at room temperature. The available amine groups in the gelator are responsible for rapid gelation via non-covalent interactions with Zn(II)-ion in presence of water molecules.

In this study, we have successfully developed non-volatile resistive random access memory (RRAM) devices based on Zn(II)-metallohydrogel (Zn@PEH)-mediated metal–semiconductor (MS) junctions. Our aim is to develop a versatile RRAM device with different configurations for applications in neuromorphic computing and data-driven applications like the Internet of Things (IoT), 5G connectivity etc.

On the other hand, we have also focused novel computing architectures to solve the von Neumann bottleneck. It can be solved by introducing the new architecture concepts of neuromorphic computing and in-memory computing. Our research have focused on resistive switching memory (RRAM) device which can be used as a memory and computing element at the same time. Recently, it has an advantage of the RRAM ability to add multiple applied pulses, which acts as a logic gate. RRAM logic gates are based on the conditional switching of one or more output RRAM which depends on the states of the input RRAM devices and the applied voltage amplitudes. These devices can be applied as synaptic elements^65,66^. They must have specific characteristics such as high ON/OFF ratio, multilevel functionality, and strong compatibility to optimize synaptic or neuron elements. Since, this kind of device is non-volatile and nonlinear; it is more advantageous to use in brain synaptic networks, logic circuits and other flexible electronics devices. Our work provides a brief overview of them. Additionally, we investigate the potential applications of metallohydrogel based memristors in crossbar array by designing logic gate circuit. Consequently, memristor-based logic gate circuit opens up a new approach for development of new advance technology in memory application.

**Figure 1:**
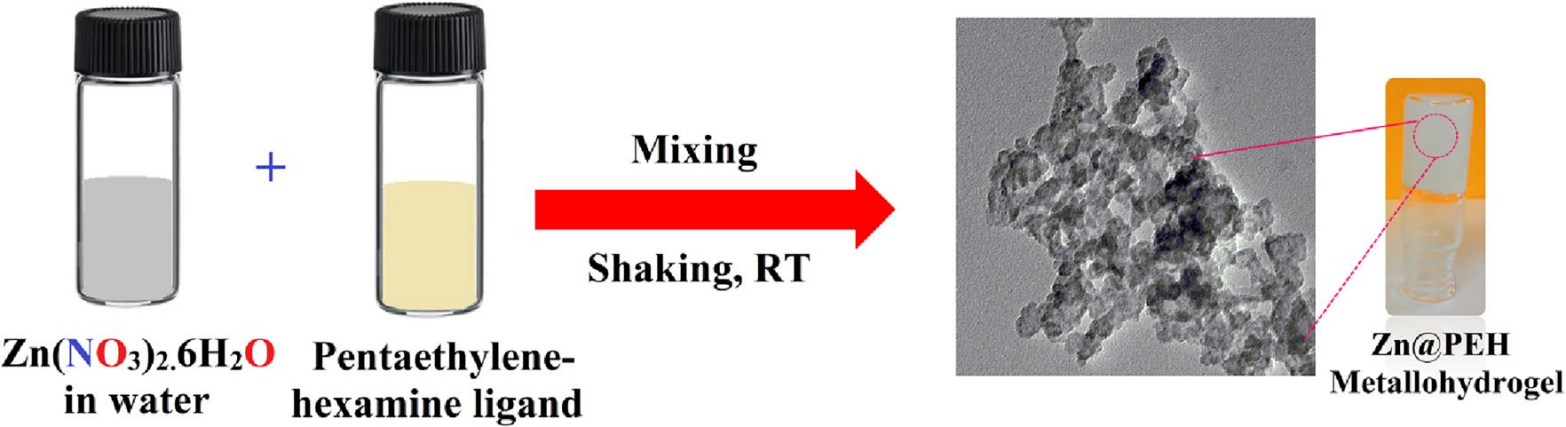
Schematic representation of the probable gelation mechanism in Zn(II)-metallohydrogel (Zn@PEH).

**Figure 2:**
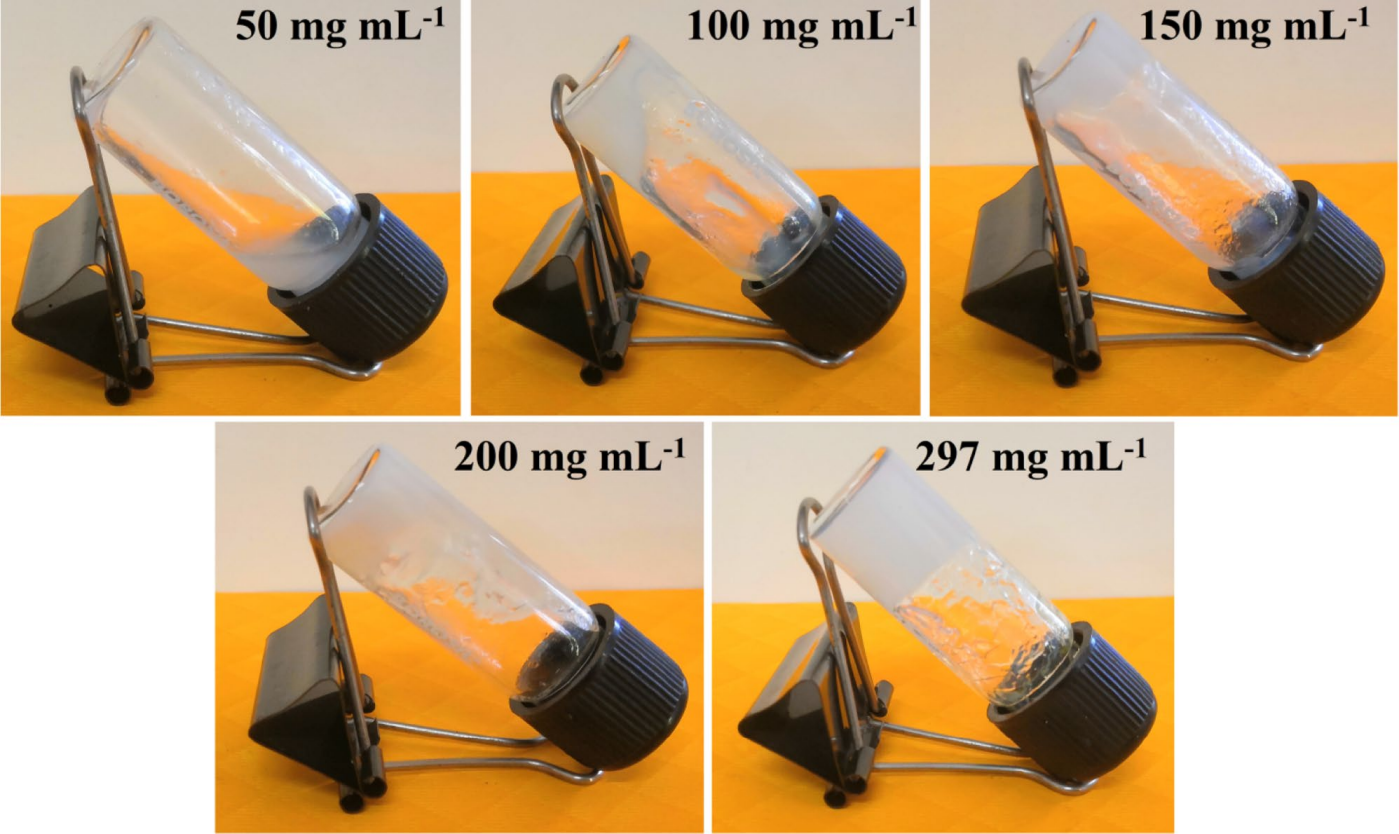
Pictures showing the determination MGC of Zn(II)-metallohydrogel (297 mg mL^-1^) by varying concentrations.

In this work, a 500µL colourless aqueous solution of Zn(NO_3_)_2_.6H_2_O (0.297 g, 1 mM) and 500µL pentaethylenehexamine were mixed at one-shot in a 5 ml glass vial at room temperature under ambient conditions. White colour stable Zn(II)-metallohydrogel (i.e. Zn@PEH) was generated after the gentle mechanical shaking of the vial (Fig. 1). Figure 1 shows probable synthetic representation of Zn@PEH and inverted image of stable Zn@PEH. Minimum critical gelation (MGC) of Zn@PEH was calculated by varying the concentrations of Zn@PEH metallohydrogel from 50 to 297 mg mL^-1^. The Zn@PEH metallohydrogel was generated efficiently at the concentration 297 mg mL^-1^ (Fig. 2). The gel to sol transition temperature (*T*_*gel*_) of Zn@PEH was 210 °C.

## Results and discussion

### Self-healing performance of Zn@PEH metallohydrogel

**Figure 3:**
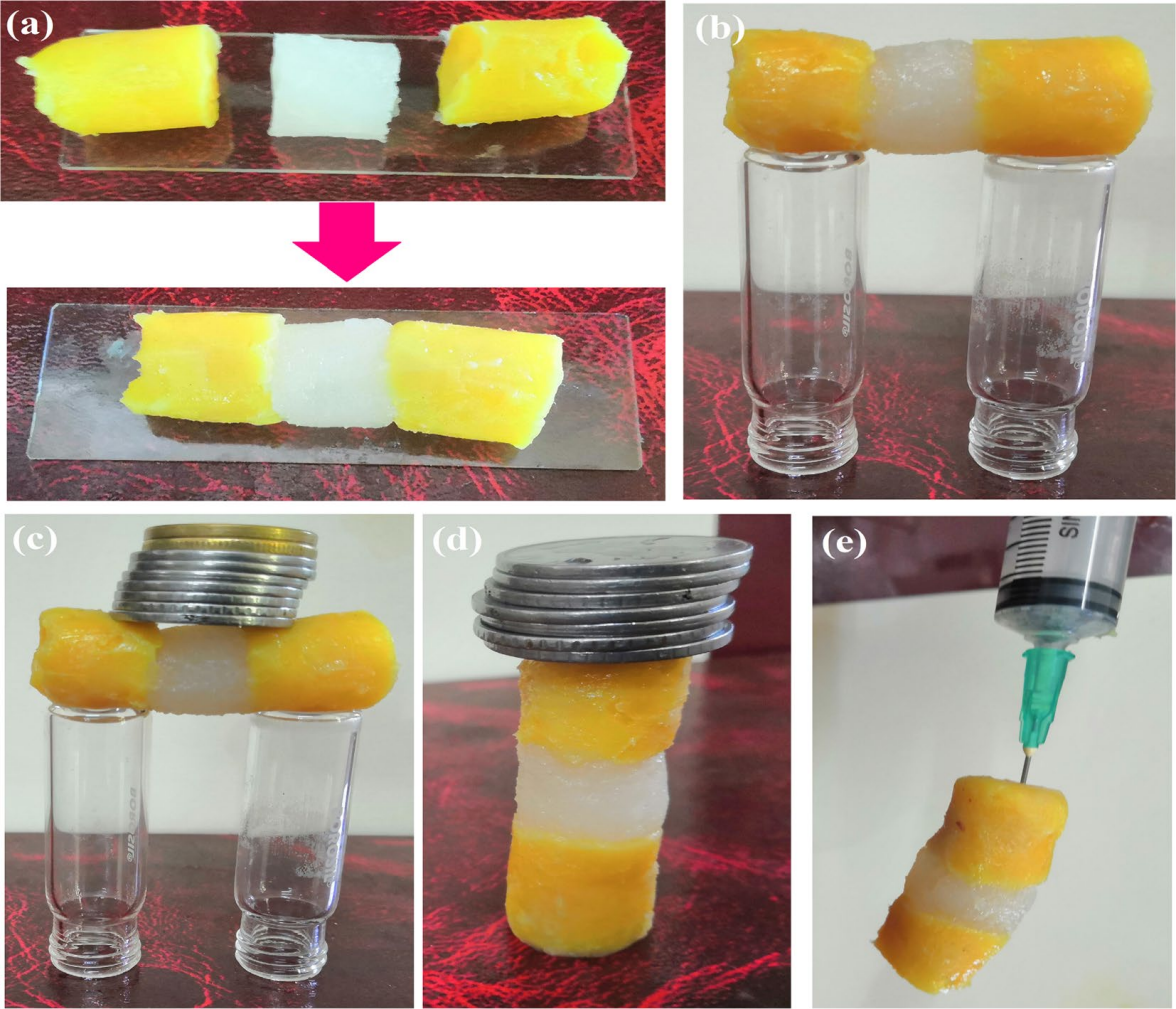
(**a**,**b**) Self-healing and self-supported monolith bridge constructed by cylindrical blocks of pure Zn@PEH metallohydrogel and Fluorescein dye doped Zn@PEH metallohydrogel; (**c**,**d**) load-bearing performance of horizontally and vertically cured monoliths; (**e**) Alternative system of self-healing Zn@PEH gel against gravity.

To perform the self-healing test we have prepared monolith of pure Zn@PEH hydrogel and fluorescein dye doped Zn@PEH hydrogel following the described synthetic method (Fig. 3). Blocks made of pure Zn@PEH and fluorescein dye doped Zn@PEH were cut to approximately the same dimensions (i.e., ~ 1 to 2 cm) and placed back into contact by slight pressing and completely welded to each other in < 5 min. (Fig. 3a). The healed blocks showed a strong superiority capable of free standing without any external support (Fig. 3b). Consequently, the addition of dye cannot affect the strong self-healing properties of Zn@PEH metallohydrogel. (Fig. 3). Interestingly, the monolith bridge showed extremely self-resisting performance with excellent load-bearing capacity (~ 55 g) (Fig. 3c). Even, the cured monolith bridge showed good vertical load carrying capacity (~ 50 g) (Fig. 3d). The stability of monoliths after curing against gravity is also revealed in Fig. 3d.

### Injectability of the self-healing Zn@PEH metallohydrogel

**Figure 4:**
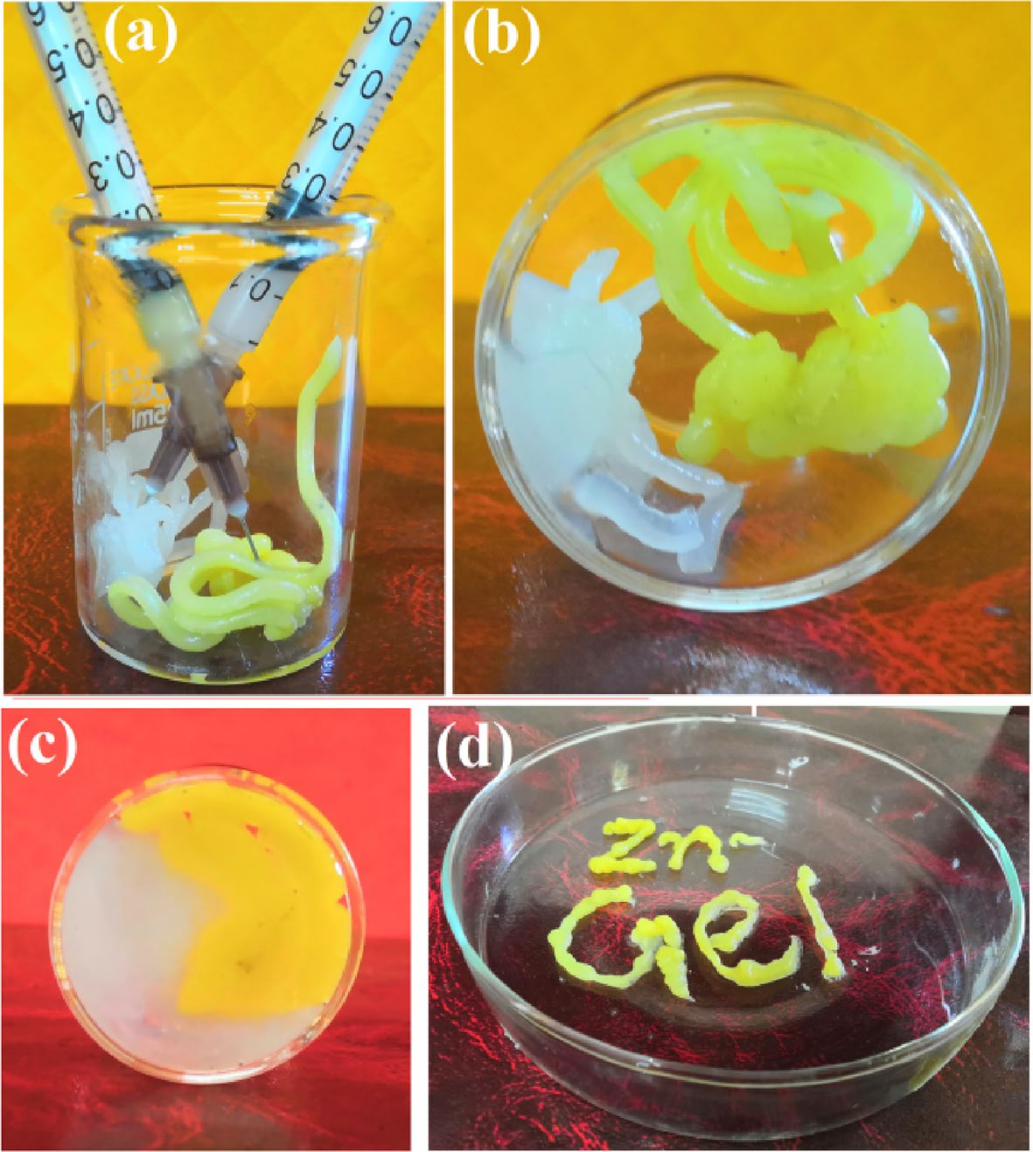
(**a**) The injectable process of the self-healing Zn@PEH metallohydrogel; (**b**,**c**) injected separately from needles and compacted at the bottom of the beaker; (**d**) injectable gel used as ink.

The self-healing Zn@PEH metallogel could also be used as an injectable metallogel. The self-healing Zn@PEH metallohydrogel can be injected after gelation. For better picturing of the injectability of Zn@PEH, pure Zn@PEH and fluorescein dye stained Zn@PEH gels were injected into a 10 mL beaker from two different needle tubing and compacted in the bottom of the beaker (Fig. 4a-c). Additionally, a sketch was drawn to show the injectibility and use of Zn@PEH gel as an ink (Fig. 4d).

### Rheological analysis

**Figure 5:**
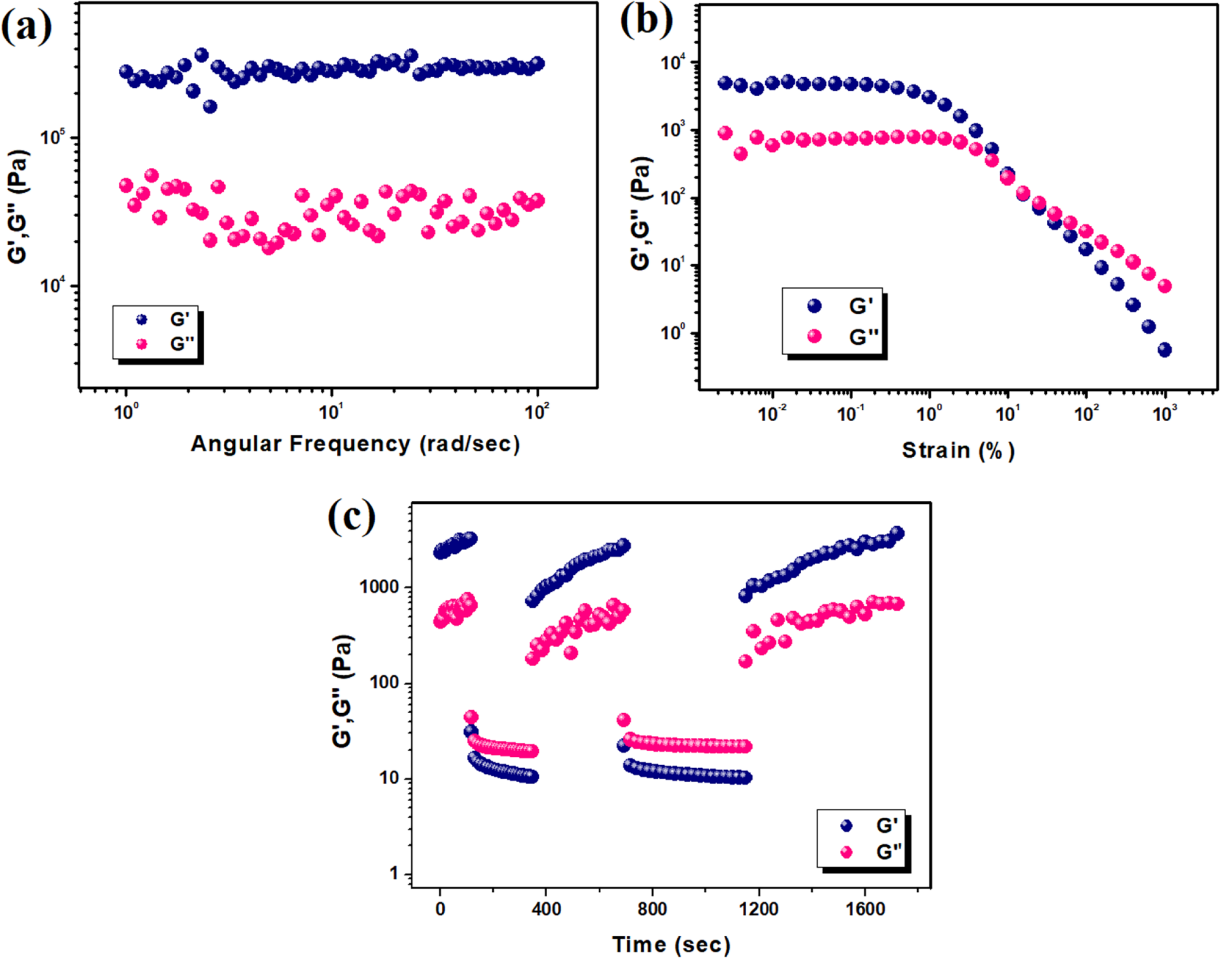
(**a**) Angular frequency vs. storage modulus (*G′*) and loss modulus (*G″*) plot of Zn@PEH metallohydrogel; (**b**) Strain-sweep experiments of Zn@PEH metallohydrogel performed at a constant frequency of 6.27997 rad/sec; (**c**) Thixopropic measurement of Zn@PEH metallohydrogel.

Rheological characterization of soft materials like supramolecular gels are crucial in terms of their applications in various purposes The mechanical properties of Zn(II)-metallohydrogel was characterized through rheological experiments, where larger values of storage modulus (*G′*) than loss modulus (*G′′*), authenticated the viscoelastic semi-solid nature of gel materials (Fig. 5). For a material to be considered as a gel, the condition *G′*(*ω*) > *G″*(*ω*) [where, *G′*(*ω*) ≈ *ω°* and the angular frequency is ω] has to be satisfied. In order to do that, we have initially performed frequency sweep test of the matallogel on a Zn(II)-metallohydrogel at a fixed concentration of Zn(NO_3_)_2_.6H_2_O (i.e. [Zn(II)] = 297 mg mL^-1^). The magnitude of storage modulus (*G'*) was found to be much larger than the corresponding loss modulus (*G′′*), irrespective of the applied angular frequency with a *G'/G''* ratio ~ 1.05, indicating viscoelastic nature of the metallogel (Fig. 5a). Further, amplitude sweep experiment was carried out that depicted linear viscoelastic region up to stain of ~ 1%. The cross over between *G'* and *G''* was observed at a strain of ~ 10%, indicating gel to sol transition (Fig. 5b).

Finally, thixotropy test was performed to check the self-healing ability of the metallogel. At low strain (0.01%), the viscoelasticity of the gel remains intact. When the strain increased suddenly to ~ 100%, the viscoelastic nature of the soft material abolished almost immediately (Fig. 5c). The sol to gel transition happens within seconds upon decreasing the strain back to 0.01%, thus clearly proving self-healing nature of the metallogel. Such low to high strain cycle was repeated, which showed that the viscoelasticity was recovered after each cycle (Fig. 5c).

### Field emission scanning electron microscopic (FESEM) study

**Figure 6:**
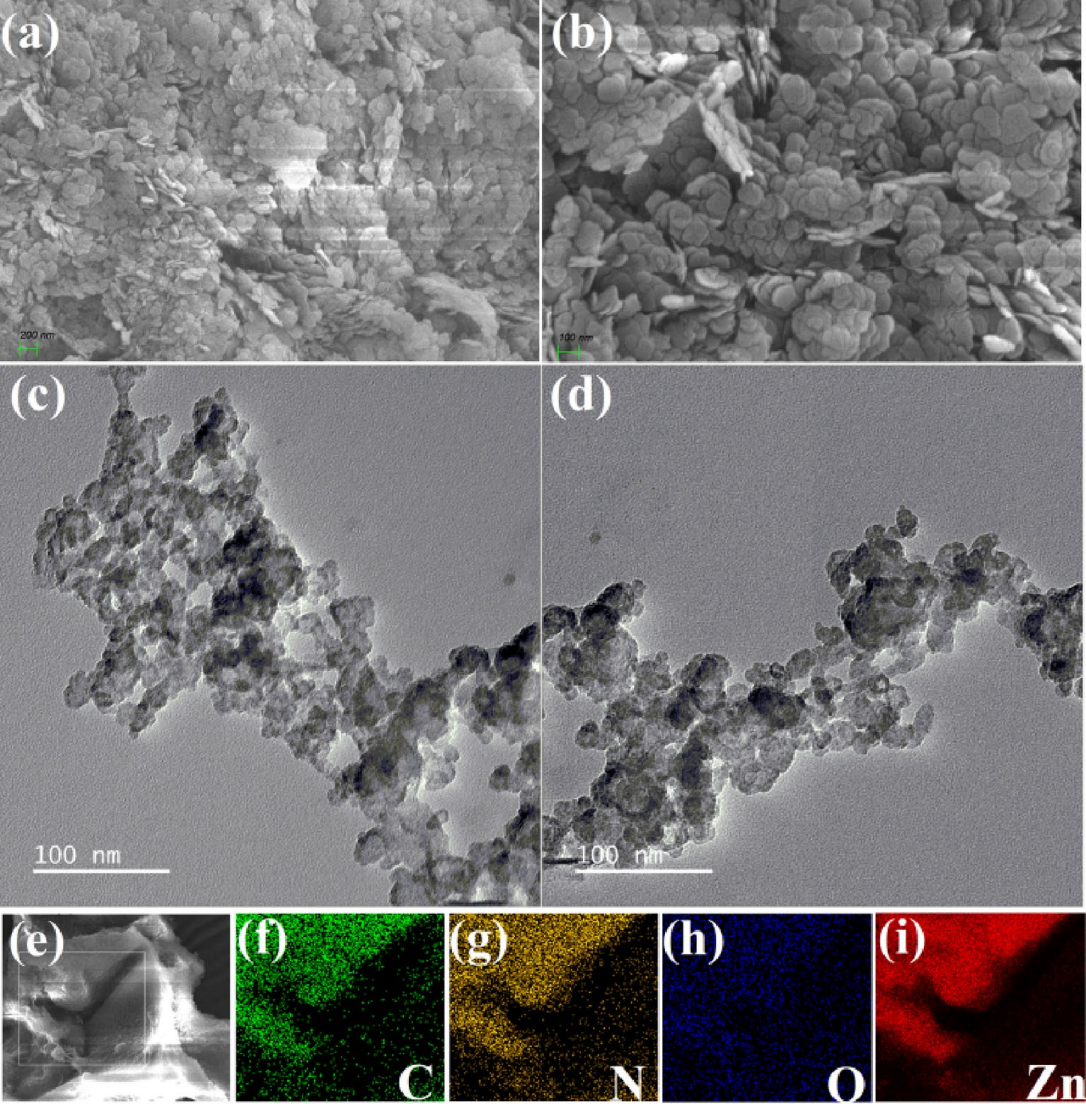
(**a**,**b**) The FESEM images of Zn@PEH metallohydrogel; (**c**,**d**) HRTEM images patterns of Zn@PEH metallohydrogel; (**e**–**i**) elemental mapping of Zn@PEH metallohydrogel showing the presence of main constituent elements C, N, O, and Zn.

The microstructal patterns of Zn@PEH metallohydrogel were characterized through FESEM and TEM structural analysis. The FESEM pattern of the Zn@PEH metallohydrogel showed pebble like hierarchical architecture (Fig. 6a,b). Hierarchical morphologies of Zn@PEH metallohydrogels observed under FESEM can be formed by the combination of Zn(NO_3_)_2_.6H_2_O and pentaethylenehexamine gelators in water via rapid mixing through a variety of non-covalent interactions like hydrogen bonding patterns, appropriate metal–ligand coordination, electrostatic connections, hydrophobic interactions. High resolution TEM images show agglomerated hemi-spherical shape of Zn@PEH metallohydrogel samples about ~ 100 nm (Fig. 6c,d) size. EDX elemental mapping confirms the presence of C, N, O and Zn elements of Zn(NO_3_)_2_.6H_2_O, pentaethylenehexamine gelator and water solvent, responsible in Zn@PEH metallohydrogel networks (Fig. 6e–i).

### Fourier transform infrared (FT-IR) spectroscopic study of Zn@PEH metallohydrogel

**Figure 7:**
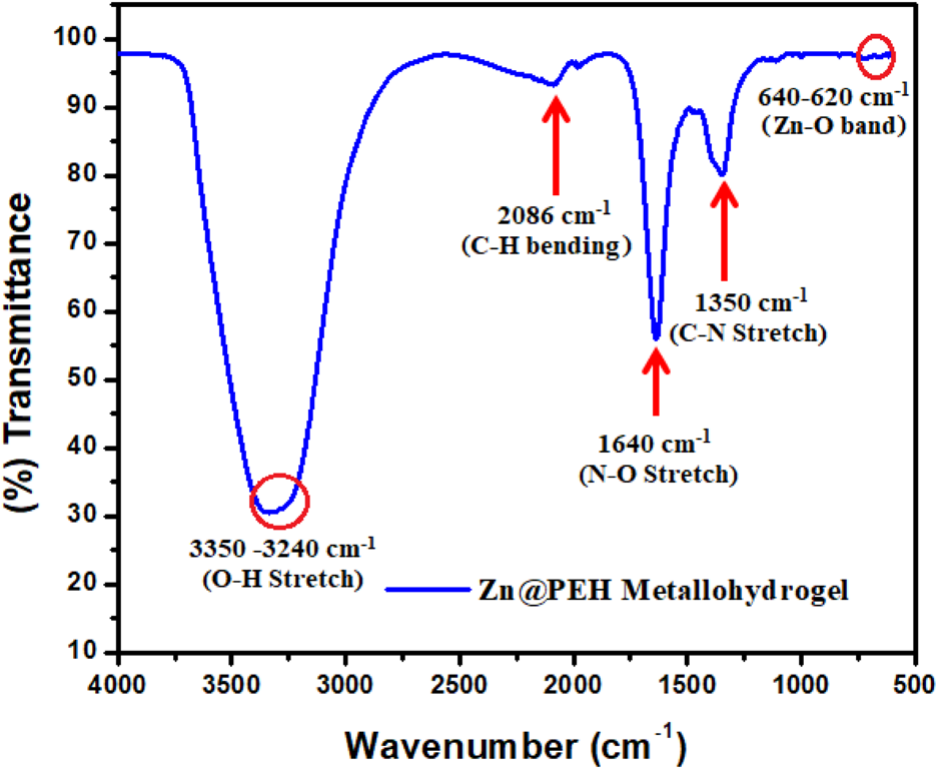
FT-IR spectra of the xerogel form of Zn@PEH metallohydrogel.

Fourier transform infrared (FT-IR) spectra of Zn@PEH metallohydrogel in its xerogel form exposed the main responsible interaction of pentaethylenehexamine with Zn(II)-ion in water medium for Zn@PEH Metallohydrogel formation (Fig. 7). The significant spectral absorption bands are at seen at 3350–3240 cm^-1^, 2086 cm^-1^, 1640 cm^-1^, 1350 cm^-1^ and 640–620 cm^-1^. The wide broad peaks in the region of at 3350–3240 cm^-1^ are due to O–H stretching vibrations. Vibrational modes at 2086 cm^-1^ are attributed to C-H bending. The vibrational modes associated with the peaks at 1640 cm^-1^, 1350 cm^-1^ and 640–620 cm^-^^1^are N–O stretching, C–N stretching, and Zn–O band respectively (Fig. 7).

### Optical characterization

**Figure 8:**
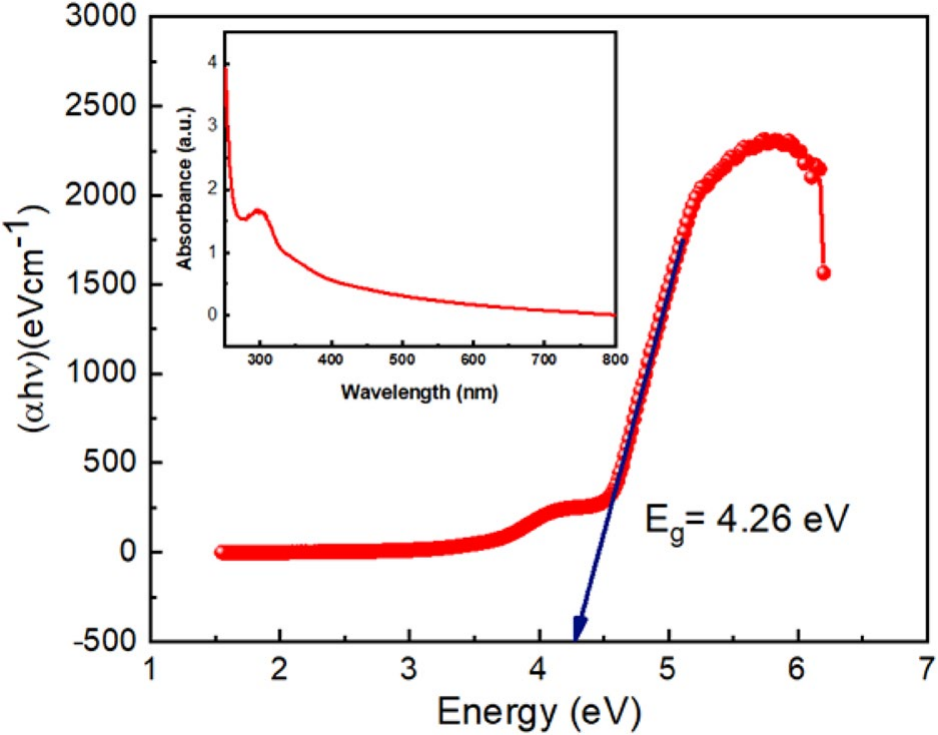
UV–Vis absorption spectra (inset) and Tuac's plots for Zn@PEH metallohydrogel.

Figure 8 illustrates the Tuac’s Plot using UV–vis absorption spectra to investigate the optical characteristics of the prepared metallogel Zn@PEH. The wavelength range for the optical measurement was set between 250 and 800 nm (refer to the UV–vis absorption spectra). Tauc's equation, represented by (Eq. 1), was used to determine the optical bandgap (E_g_) of the Zn@PEH metallogel from the UV–vis spectrum:

1 $$\left( {\alpha {\text{h}}\nu } \right)^{{\text{n}}} = {\text{ A }}\left( {{\text{h}}\nu - {\text{E}}_{{\text{g}}} } \right)$$

Where absorption coefficient, band gap, Planck's constant, and frequency of light are denoted by α, E_g_, h, and v respectively. The exponent "n" is a constant in electron transition processes. A constant with the value of 1 is used as the value of "A." The direct optical band gap was calculated using n = 2. By extending the linear region of the plot (αhν)^2^ vs hν (Fig. 8) to the region where absorption vanishes, we were able to calculate the direct optical band gap (E_g_), which is estimated to be 4.26 eV.

### Device fabrication

In this study, to study the electrical behaviour of the synthesised material, we developed a lateral Schottky diode-like structure based on a metal–semiconductor junction in the ITO (Indium Tin Oxide)/Zn@PEH/ITO configuration (referred to as **device 1**). To create the junction device, we grew a thin film of as-synthesised Zn@PEH using the Doctor's blade technique on a glass substrate covered in ITO, and then we annealed the film to remove the solvent. ITO is strongly conducting and optically transparent in the visible spectrum of the electromagnetic spectrum and it is advantageous for studies involving photo-excitations.

RRAM (Resistive Random Access Memory) devices based on Zn@PEH metallohydrogel were created using vertically stacked sandwiched structures of ITO/Zn@PEH/Cu (named **device 2**) and Cu/Zn@PEH/Cu (named **device 3**). Both designs were developed with clean bottom electrodes, after that Zn@PEH metallogel was deposited on the substrate, and then the top electrode was placed on the sample.

The device structures are given by the following Table 1.

**Table 1:** Different devices with their structures.

Device no	Device structure
Device 1	ITO/Zn@PEH/ITO
Device 2	ITO/Zn@PEH/Cu
Device 3	Cu/Zn@PEH/Cu

### Electrical characterization

**Figure 9:**
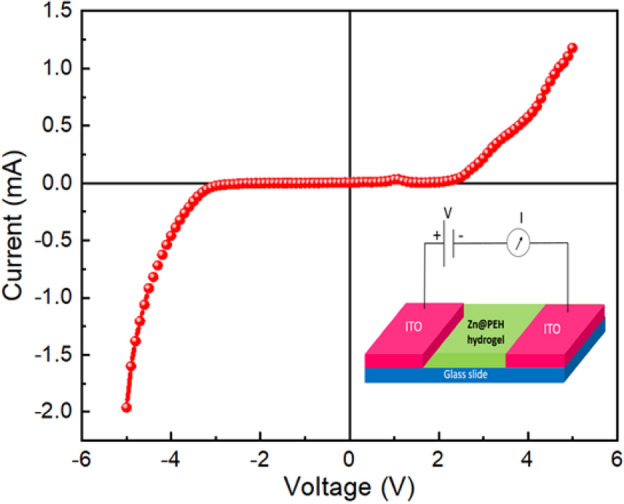
Schematic diagram of Zn@PEH metallohydrogel-based device (ITO/Zn@PEH/ITO) (inset) and I-V curve for (ITO/Zn@PEH/ITO) device at room temperature in the linear scale.

By verifying the existence of a band gap, we were able to further comprehend the semiconductor nature of the Zn@PEH metallohydrogel in the thin film geometry by examining its charge transport characteristics. In the voltage range of (− 5 V to + 5 V), where the I-V curve of device 1 is displayed in Fig. 9 on a linear scale, there is no detectable conduction. After this, the current starts to rise quickly in both the positive and negative polarity of the applied voltage. Then the I-V data were analysed, and the key diode parameters were taken from the non-linear Schottky diode's I-V curve using the thermionic emission theory (TE Theory). Cheung proposed this technique to analyse the conduction behaviour of such systems ^52.^ The measured I-V curve has been evaluated numerically using the following equations (2) and (3):

2 $$\mathrm{I }= {I}_{0} exp\left(\frac{qV}{\eta {k}_{B}T}\right)\left[1-exp\left(\frac{-qV}{\eta {k}_{B}T}\right)\right]$$

3 $${I}_{0}= A {A}^{* }{T}^{2 }{\text{exp}}\left(\frac{-q{\phi }_{B}}{{k}_{B}T}\right)$$

Here, I_0_ = Saturation Current, q = Electronic Charge, k_B_ = Boltzmann’s Constant, T = Temperature, V = Applied voltage, A = Effective diode area, η = Ideality factor, Φ_B_ = Barrier potential height, R_S_ = Series resistance, A* = Effective Richardson constant which is considered as 32 AK^-2^ cm^-2^ for this device.

**Figure 10:**
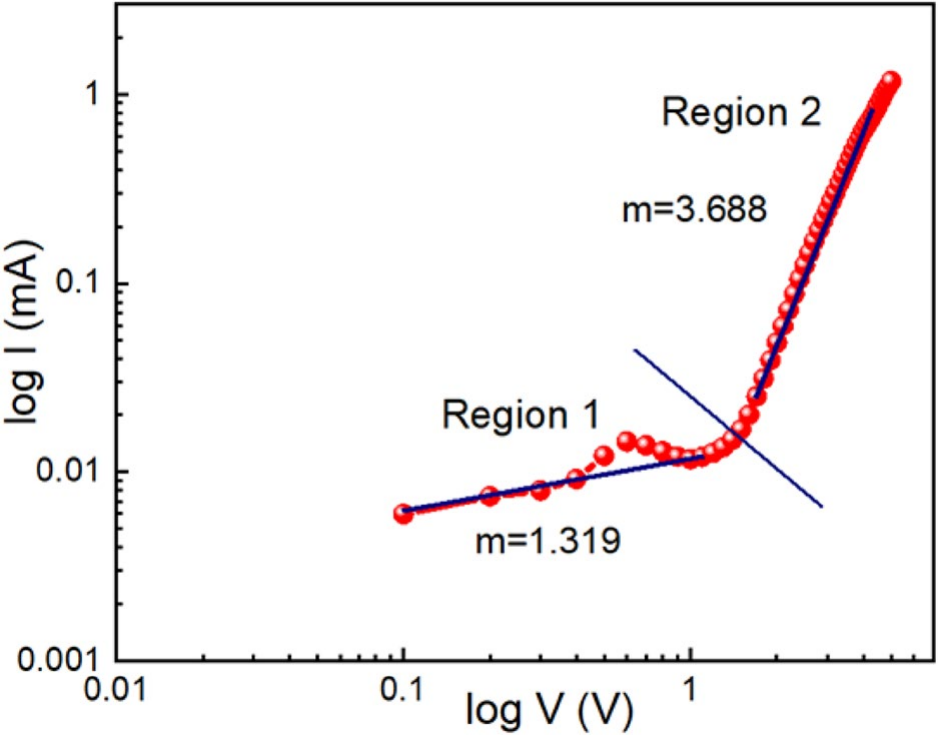
I-V curve of ITO/Zn@PEH/ITO based device in logarithmic scale.

To fully understand the conduction mechanism, we plotted the log I vs. log V graph, which is shown in Fig. 10. On log scale, this I-V graph is divided into two voltage zones with unique slopes. Region 1 which corresponds to lower voltage region, has a slope of 1.319, and here, the current conducts by Ohmic conduction. When the voltage is increased, the slope in region 2 is 3.688, indicating that the device follows a space charge-limited conduction mechanism. We may conclude that this mechanism is guided by the trap-filled Space Charge Limited Conduction (SCLC) technique as the slope is more than 2.

4 $$\frac{dV}{d({\text{ln}}I)}= \left(\frac{\eta {k}_{B}T}{q}\right)+I{R}_{S}$$

5 $$H\left(I\right)=V- \left(\frac{\eta {k}_{B}T}{q}\right){\text{ln}}\left(\frac{I}{A{A}^{*}{T}^{2}}\right)$$

6 $$H\left(I\right)=I{R}_{S}+ \eta {\phi }_{B}$$

**Figure 11:**
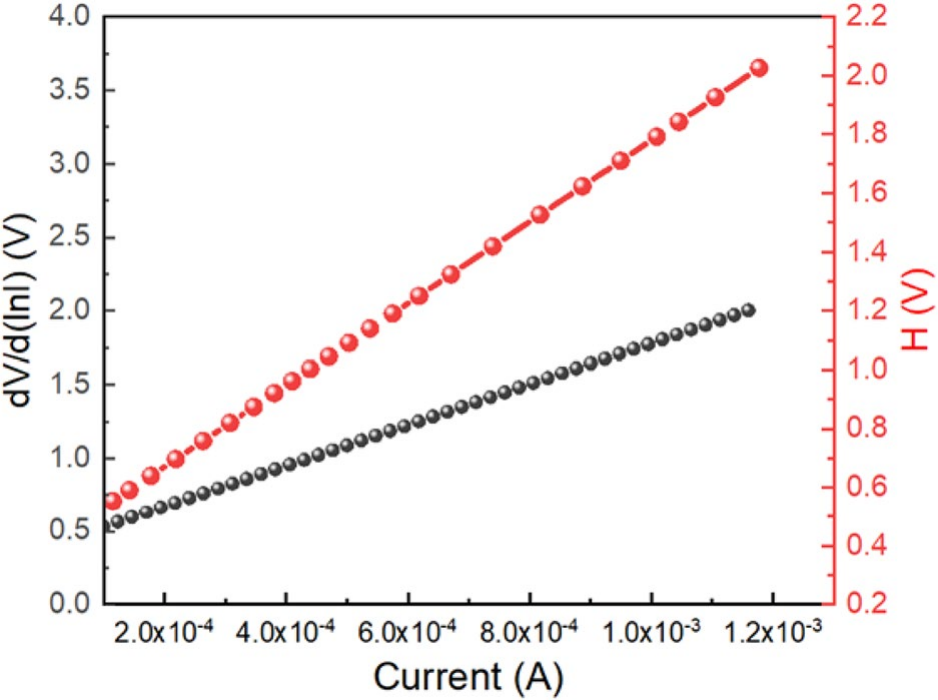
dV/d(ln I) vs. I graph and the H vs. I graph for ITO/Zn@PEH/ITO device.

Equations (4) through (6) were used to compute the series resistance, ideality factor, and barrier potential height. These equations were derived directly from Cheung's theory. To ascertain the diode specifications of the device 1, we plotted the dV/d(ln I) vs. I graph and the H vs. I graph, as shown in Fig. 11. We calculated the barrier height from the intercept of the H vs. I graph and also determined the ideality factor ($\eta$) from the intercept of the dV/d(ln I) vs. I graph. Our diodes' ideality factor ($\eta$) was calculated at a value of 15.2730, which is higher than the ideal value ≈ 1. The divergence of actual behaviour may be caused by series resistance at the interface, the existence of interface states, and the presence of inhomogeneities in the Schottky barrier.

For device 1, a barrier height (${\phi }_{B}$) of 0.0258 eV was calculated. It is so evident that the built in diode has a lower barrier potential height and a higher ideality factor, both of which are required for a Schottky diode. The series resistance (≈1385.81Ω) was calculated from the slope of the dV/d(ln I) vs. I graph as well as the H vs. I graph. It can be concluded that such diode layouts can be helpful for semiconductor electronics design after accounting for all of the measured properties.

**Figure 12:**
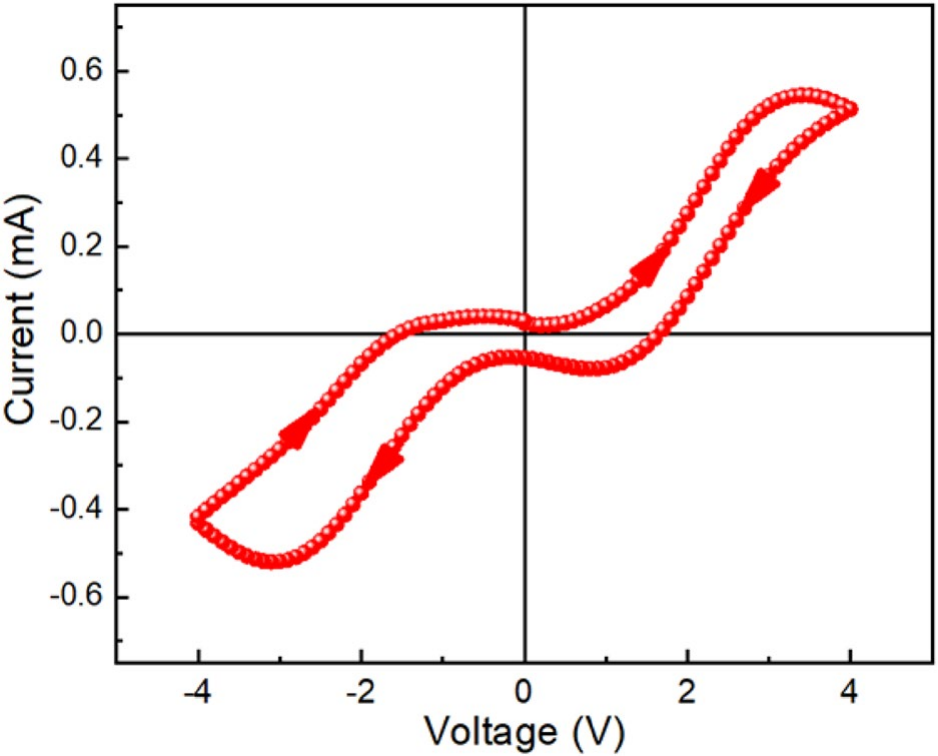
I-V curve of ITO/Zn@PEH/ITO based device where hysteresis behaviour is shown.

For device 1, while we applied the voltage over a complete cycle, a memristive nature in the I-V curve was observed as shown in Fig. 12. This is signature of resistive switching behaviour which is observed while the voltage was switched from 0 V → 4 V → − 4 V → 0 V. It suggests that the RS behaviour is observed even in the lateral conduction geometry of the system.

**Figure 13:**
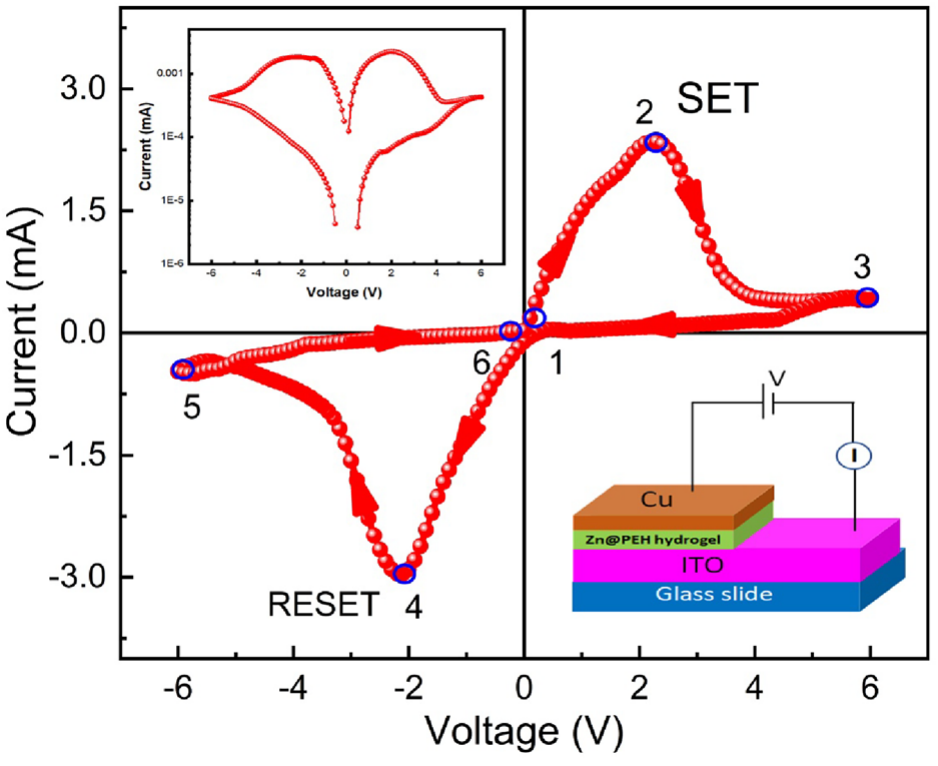
Schematic representation of a glass/ITO/Zn@PEH/Cu-based device (right: inset) and I-V characteristics plotted on a linear scale for the same device (Points 1 to 2 indicates current increases linearly, 2 to 3 indicates current decreases rapidly, 3 to 4 indicates reversing of polarity with continueous flow of current, 4 to 5 indicates current increases abruptly, 5 to 6 indicates current increases continuously up to 0 V) and I-V Curve of glass/ITO/Zn@PEH/Cu based device in semi-logarithemic scale(left: inset).

The resistive switching behaviour of the Zn@PEH metallohydrogel-based heterostructure is investigated further using the IV measurements for device 2, where ITO acts as a bottom and Cu acts as a top electrode (see Fig. 13(right: inset)). Compliance current (CC) is set at 100 mA prior to the start of any tests in order to prevent any leakage contribution to the I-V measurements. In Fig. 13, I-V curve of the device is shown on a linear scale. In this case, the I-V measurements are carried out in the following order: 0 V → 6 V → − 6 V → 0 V. The arrows represent the sequence of applied voltage cycling. Hysteresis is entirety exhibited by the I-V loop, which is a sign of memristor behaviour. At first, when the voltage is close to 0 V, the current increases linearly across the low-voltage zone (point 1 to point 2). Under a particular voltage (2.25 V), there is sudden decrease in current and as the applied voltage rises higher during SET process. The SET voltage (V_SET_ = 2.25 V) indicates that the system now moves from a higher resistance state (HRS) to a lower resistance state (LRS). At point 2, the device remains in the ON state. After the voltage drops, the current begins to drop quickly from point 3 to 4 and the device switches off at point 4 during RESET process at V_RESET_ = -2.11 V. On continuing the cycling in the negative polarity until the current starts to increase, the device reaches an LRS from point 4 to 5. Then the device goes back to the HRS at point 6. The RESET method indicates how the system switches from LRS to HRS. As a negative voltage is needed to get the sample back to its previous resistance state, this is an important evidence of the sample's bipolar resistive switching behaviour. In Fig. 13, the I-V characteristics of device 2 are shown on a semi-logarithmic scale (left: inset). The formation and rapture of conductive filament-type structures results in an increase and decrease in current which tends to switch the device between the ON and OFF states. This mechanism is explained at a later part of this work.

**Figure 14:**
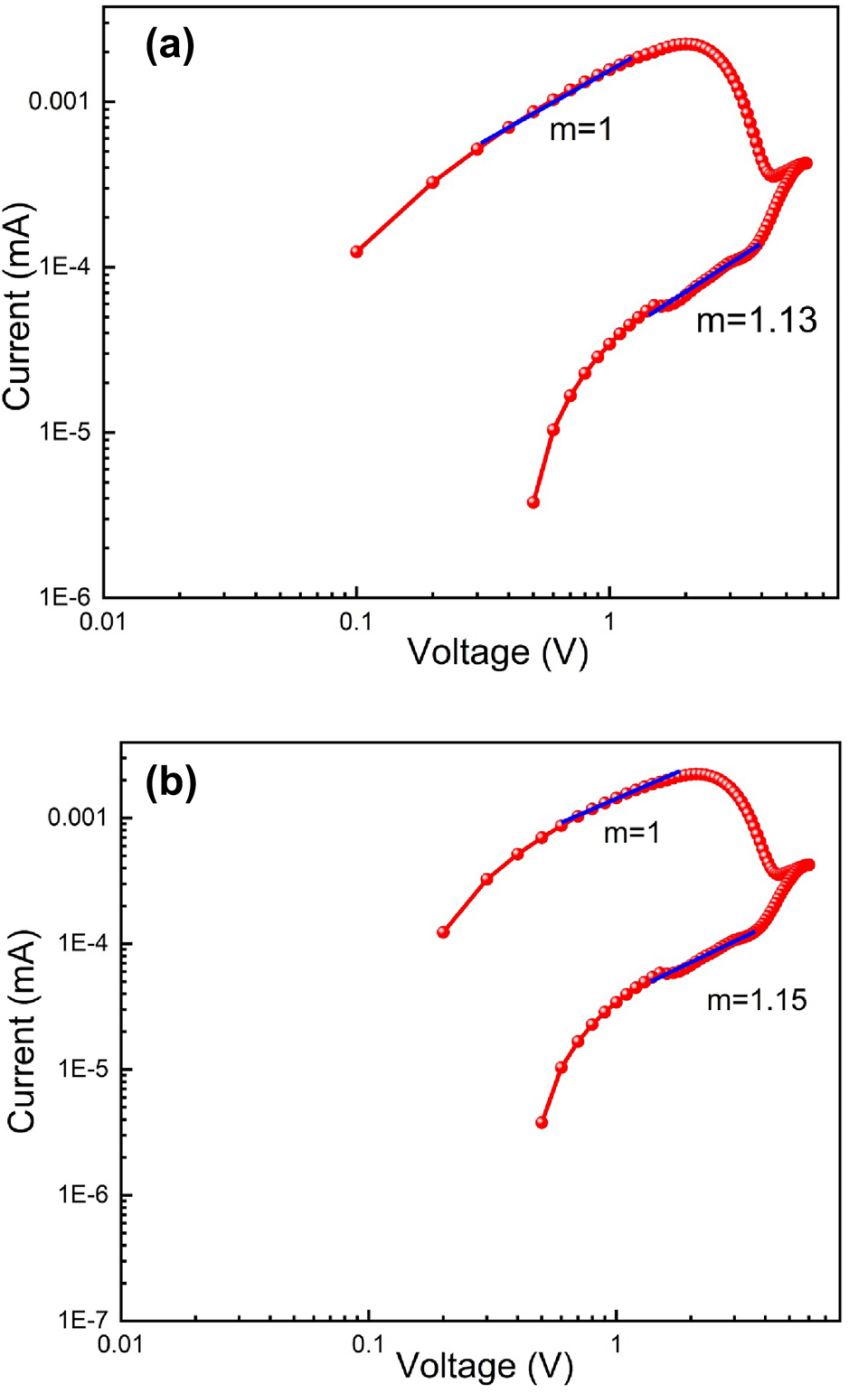
(**a**) I-V Curve of glass/ITO/Zn@PEH/Cu based device on a logarithmic scale in SET process. (**b)** I-V Curve of glass/ITO/Zn@PEH/Cu based device on a logarithmic scale in RESET process.

We have fitted the I-V curve in logarithmic scale (Fig. 14a) throughout the SET procedure to acquire a better understanding of the conduction mechanism and the charge transport mechanism of device 2. In this case, we concluded that the current follows the Ohmic nature of conduction and varies linearly in the voltage range of 0 V to 2 V with a slope of m = 1. The RESET process has also revealed similar behaviour (Fig. 14b). At lower voltage region from 0 to 2 V, we have observed that it behaves with Ohomic nature with slope of m = 1. But at higher voltage region, it is also following Ohomic conduction behavior with slope of m = 1.15.

**Figure 15:**
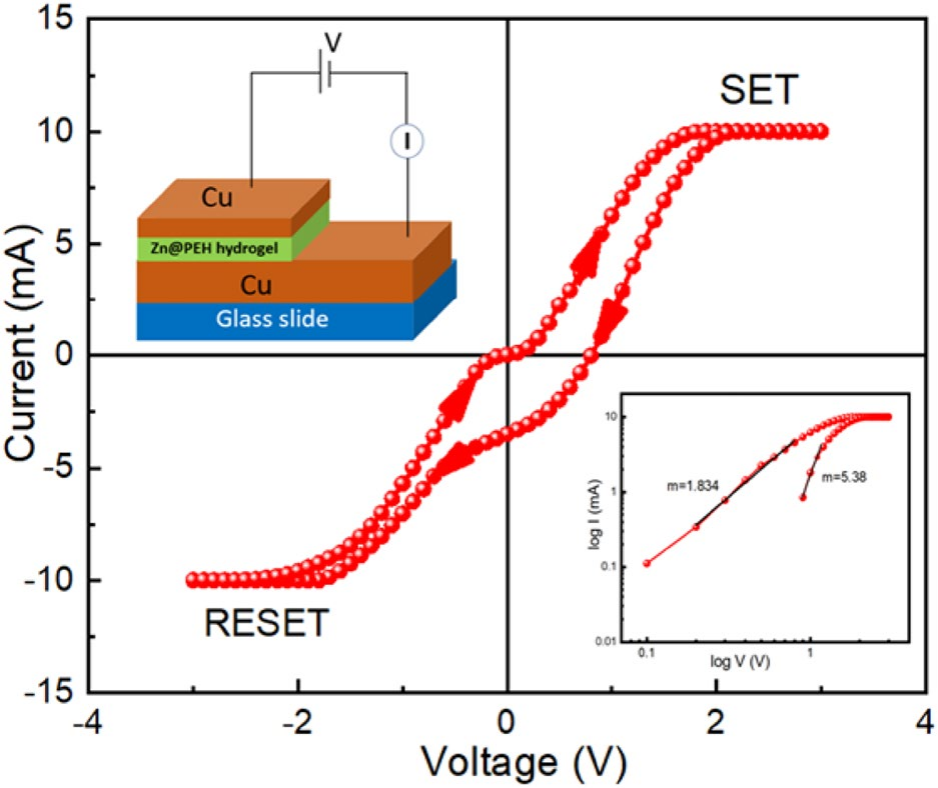
Schematic representation of a glass/Cu/Zn@PEH/Cu-based device (left: inset) and I-V characteristics plotted on a linear scale for the same device and I-V curve for same device on logarithemic scale in SET process (right: inset).

In the device 3(Cu/Zn@PEH/Cu), we have used Cu as both the top and bottom electrode to understand the robustness of the resistive switching properties of the metallohydrogel for different electrode materials. We have measured I-V characteristics for Cu/Zn@PEH/Cu as shown in Fig. 15 via DC sweeping measurements. In this case, the I-V measurements followed this sequence: 0 V → 3 V → -3 V → 0 V. I-V characteristic shows pinched hysteresis^60^ which is a sign of memristor behaviour. Figure 15 illustrates the I-V characteristics when a 5 V bias is swept in steps of 0.1 V within ± 3 V range. Though it is not properly following the ideal memristor^62,63^ behavior, our device exhibited a bipolar switching behavior with a non-zero crossing that occurred at 0.8 V as shown in Fig. 15 which indicates the presence of parasitic capacitance^61^ in our device. During SET process the device goes from HRS to LRS at 1.96 V and it remains in the ON state until we apply negative voltage to return to its previous resistance state. Similarly, during RESET process the device goes from LRS to HRS at -1.74 V and it remains in the OFF state. Consequently, non-zero crossing has been seen in our device Cu/Zn@PEH/Cu in the low voltage region, where it can be defined by an electrode-induced memcapacitive effect. Double-crosspoint hysteresis is not seen in conjunction with non-zero crossing. Although the exact nature of this capacitance is unknown, it has been suggested that it may be caused by the device architecture, which resembles a capacitor structurally and usually consists of two parallel plates separated by a metallogel in a vertically layered structure. When there is a large gathering of positive and negative ions at the interfaces between the electrodes and the functional layer, the capacitance effect may be justified. So, for the presence of a parasitic capacitance, this I-V response shows a capacitive behavior.

We have also fitted the I-V curve in logarithmic scale during the SET process (Fig. 15 (right:inset)) for better understanding of the conduction mechanism and the charge transport mechanism of device 3. In this case, we confirmed that the current follows the Ohmic nature of conduction and varies linearly in lower voltage region with a slope of m = 1.834. But when the voltage is increased, the slope is 5.38 which leads to space charge conduction behaviour.

**Figure 16:**
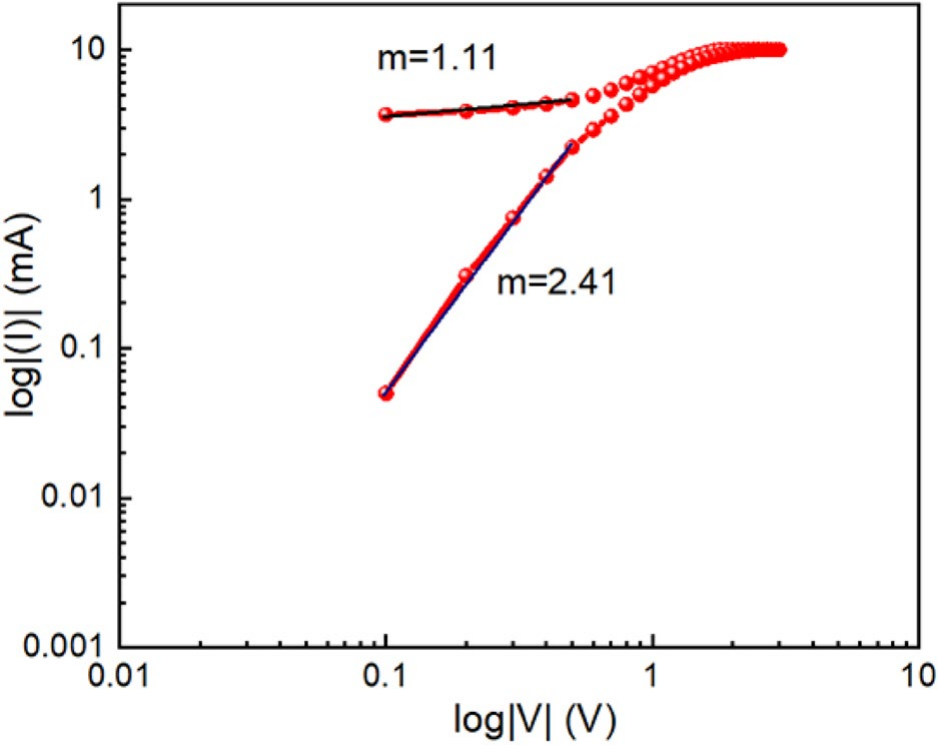
I-V Curve of glass/Cu/Zn@PEH/Cu based device on a logarithmic scale in RESET process.

During the RESET process, we have also plotted I-V curve in logarithmic scale (Fig. 16). Here, we have observed that in the lower voltage region, it follows Ohmic nature with slope m = 1.11 and at higher voltage region it is showing space charge limited conduction behaviour with slope of m = 2.41.

**Figure 17:**
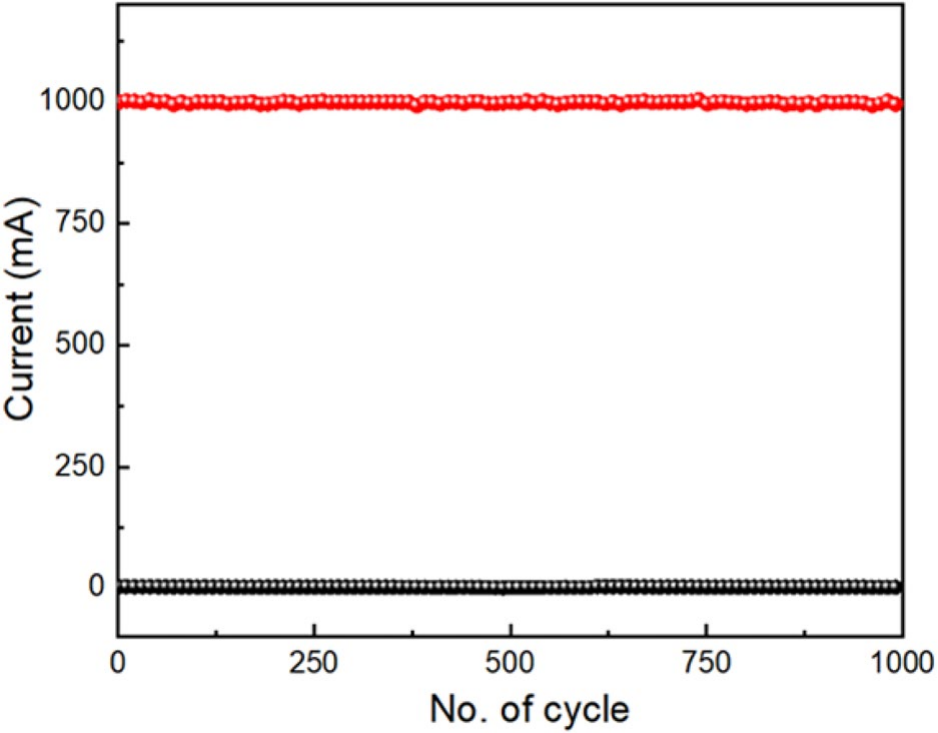
Endurance test of glass/Cu/Zn@PEH/Cu based device.

We have also conducted Endurance test over 1000 switching cycles at room temperature to better comprehend the consistency of the switching process, as shown in Fig. 17. The switching operation in device 3 is consistent up to 1000 switching cycles. According to the endurance test, the value of I_ON_ is 1005.4 mA and the value of I_OFF_ is 3.735 mA. So, the average ON/OFF ratio is 270 which indicates the robustness of the switching behaviour. It suggests that this device can maintain its memory response for a long time without suffering any degradation, which is useful for practical applications in memory circuit design at a lower manufacturing cost. We have also compared SET/RESET value, ON/OFF ratio with other materials as shown in Table 2.

**Table 2:** Comparison of SET/RESET values and ON/OFF ratio with different materials.

System	Type	V_SET_ (V)	V _RESET_ (V)	I_ON_ / I_OFF_	References
La_0.7_Sr_0.3_MnO_3_ -rGO	Bulk	9.5	− 9.7	10	^46^
LaCoO_3_- graphene	Bulk	9.8	− 8.1	40	^53^
ITO/(PF_6_)_4_/Al	Thin film	3.4	− 4.3	100	^54^
Ag/CD-MOF/Ag	Thin film	± 7.5	± 1.5	30	^55^
Cu/Mg@3AP/Cu	Thin film	2.36	− 2.93	100	^56–59^
LSMO-ZnO	Bulk	15.5	− 17	4	^67^
CuI-LSMO nanocomposites	Bulk	2	− 2	4.2	^68^
Cu/Zn@PEH/Cu	Thin film	1.96	− 1.74	270	Current work

**Figure 18:**
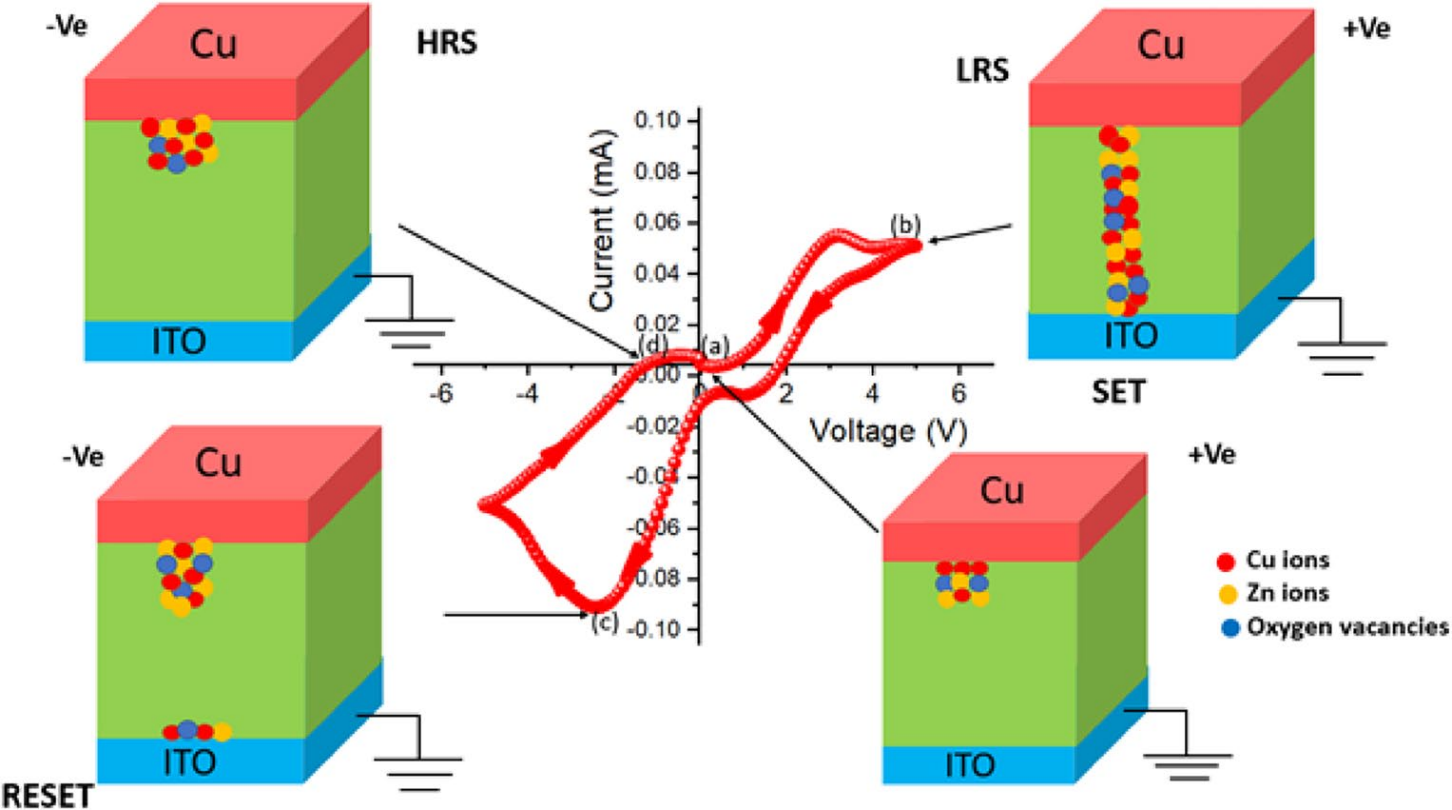
Using the conductive filament model, this diagram explains various steps involved in the resistive switching mechanism of a glass/ITO/Zn@PEH/Cu-based device. The following locations have been designated with the I-V curve: (**a**) After applying a positive voltage of 0.28 V, the ions migrated towards the intermediate layer; (**b**) at 4.88 V, Cu, Zn, and oxygen vacancies produced a conducting filament-type structure; and in step (**c**) by applying a negative voltage of − 2.35 V, Cu ions, Zn ions, and oxygen vacancies are brought back to the top electrode; and in step (**d**), all ions are gathered at the top electrode and switchted into HRS at − 0.17 V.

The physical origin of a resistive switching—process can be explained by several mechanisms, including the formation of the Schottky barrier with electrochemical migration, redox reactions, valence change memory, etc. In our case, the mobility of oxygen defects and metal cations affects the valence change memory (VCM) and electrochemical metallization process (ECM). Here, it is demonstrated that the condition of resistance is significantly affected by oxygen vacancies and metal ions (Fig. 18). For device 2, the movement of Cu ions, Zn ions, and oxygen vacancies leads to the development of conducting filaments in the semiconducting layers. By rupturing and recreating the Cu filaments that are incorporated into the semiconducting layers, we can explain the change from the HRS to LRS state. In this study, the migration of Cu ions and oxygen vacancies is primarily responsible for the resistive switching behaviour of device 2 (shown in Figure S2). We already know that in the presence of an electric field, copper atoms can ionise to produce copper ions with the formula Cu → Cu^2+^  + 2e- and they can move in the direction of applied electric field. Cu^2+^ ions, Zn ions, and oxygen vacancies move towards the intermediate layer when a positive voltage is applied, where they are converted to metallic Cu. The conductivity of this layer will increase and the concentration of Cu ions, Zn ions, and oxygen vacancies will approach towards the bottom electrode. During the SET process the device switches from HRS to LRS. The device remains in the LRS state until a sufficient voltage with the opposite polarity is provided to electrochemically dissolve the Cu filaments and oxygen vacancies for the RESET operation. When negative voltage is applied, the device enters the HRS, and its conductivity also decreases at the same time. At the end, oxygen vacancies, Zn ions, and Cu^2+^ ions return back to the top electrode. Similarly, the migration of Cu^2+^ ions, Zn ions, and oxygen vacancies are also responsible for the resistive switching behaviour of device 3. We have also observed Cu ion migration from TEM and EDAX analysis as shown in Figure S1(a)-(c).

### Comparison of SET and RESET values with different materials

**Figure 19:**
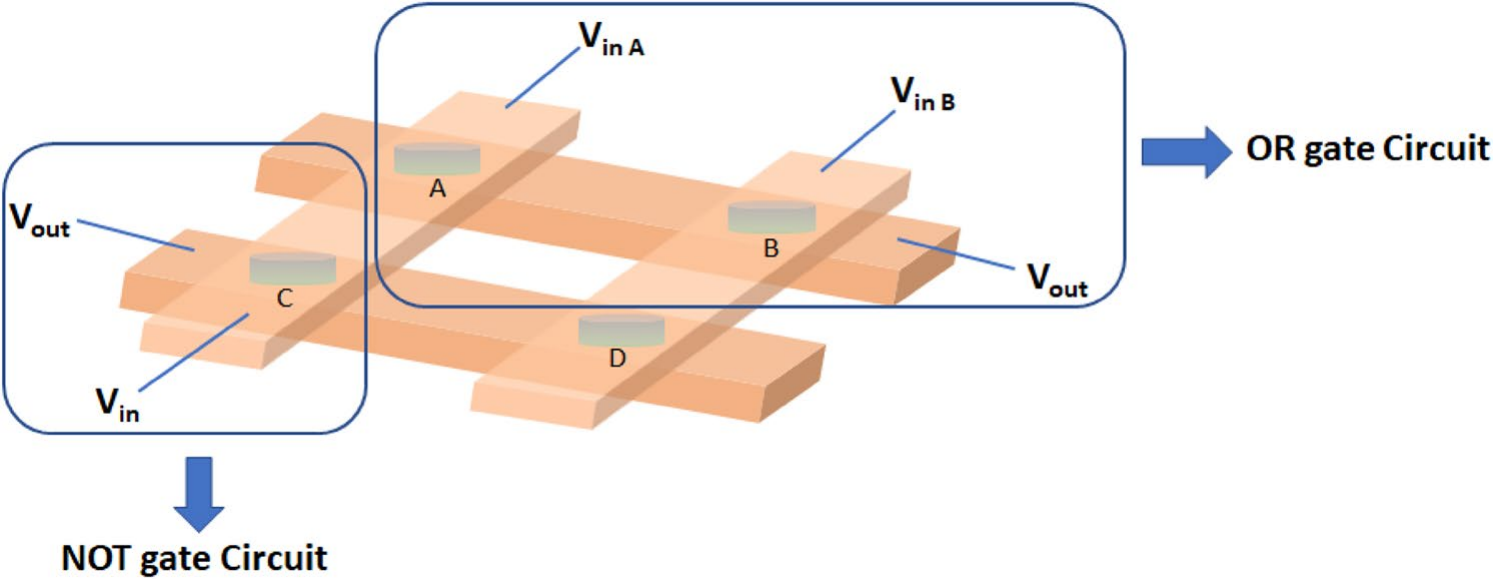
Schematic circuit diagram for OR gate and NOT gate.

In our research work, we have shown how metallogel based RRAM device in crossbar array works in memory computing using logic gate operation. In-memory computing, processing and storage both can occur at same device. Here, in this work we have prepared 2 × 2 cross bar device array based on Cu/Zn@PEH/Cu structure where Cu acts as both top and bottom electrode and four RRAM devices are denoted by A,B,C, D as shown in Fig. 19.

Now, for OR gate operation we have used two RRAM devices (A and B). If there is no applied voltage at input A and input B, then output voltage is 0.08 V which is considered as “0” logical state. When we applied 5 V at Input A and 0 V at Input B, then output voltage is 4.56 V which is considered as logical “1” state. Similarly, when we applied 0 V at Input A and 5 V at Input B, then output voltage is 4.55 V which is also considered as logical “1” state. When we applied 5 V at both the terminals, then the output voltage is also 4.58 V as logical “1” state. The truth table of OR logic gate is shown in the following Table 3. Similarly, we have designed NOT gate logic circuit (Fig. 19) using device C and it is also satisfied NOT gate truth table which is shown in the following Table 4.

**Table 3:** Truth table of OR gate.

Voltage at input A (V)	Voltage at input B (V)	Output voltage (V)	Logical state
0	0	0.08	0
5	0	4.56	1
0	5	4.55	1
5	5	4.58	1

**Table 4:** Truth table of NOT gate.

Input voltage (V)	Output voltage (V)	Logical state
0	4.51	1
5	0.03	0

The current structure can be extended further with a larger size of cross-point arrays to perform advanced logic and computing operations, which can act as a central part for in-memory computing where the computation and information storage are done at the same circuit level, as demonstrated here. In this way, RRAM based logic gate circuits using crossbar arrays will help us to explore different engineering methodologies that depends on in-memory computing principles.

## Conclusions

In summary, we successfully synthesized a stable Zn(II)-metallohydrogel by mixing zinc nitrate hexahydrate and pentaethylenehexamine in water at room temperature. This stability arises from the self-assembly of Zn(II) ions and gelator molecules through various non-covalent interactions. Rheological analysis confirmed the mechanical strength of the Zn(II)-metallohydrogel, while thixotropic data validated its self-healing property. Microstructural examination via FESEM and TEM unveiled a pebble-like hierarchical structure within the metallohydrogel. FT-IR spectroscopy characterized intermolecular interactions of metallohyrogel, while optical band-gap measurement exposed its semiconducting nature. We developed a lateral metal–semiconductor junction-based Schottky diode using Zn(II)-metallohydrogel on an ITO substrate. Furthermore, ITO/Zn@PEH/Cu and Cu/Zn@PEH/Cu based RRAM devices were fabricated in vertical configurations. Both structures exhibited bipolar resistive switching behavior which is responsible for the formation and rupture of conduction filaments. These devices show a high ON/OFF ratio of ~ 270 over 1000 consecutive switching cycles without any electrical degradation. Due to its exceptional resistive switching behavior, good endurance performance, simple fabrication, Zn(II)-metallohydrogel prefers as a promising candidate for neuromorphic applications and in advanced technological applications such as flexible microelectronics.

## Experimental section

### Materials

Zinc nitrate hexahydrate (98% reagent grade) and Pentaethylenehexamine were bought from Sigma-Aldrich and Tokyo chemical industry (TCI) chemical company and used as received. The test was all done using double-distilled water.

### Characterizations

Absorption spectroscopic data was collected through SHIMADZU UV-3101PC spectrophotometer.

Angular frequency and oscillatory amplitude sweep dependent rheological investigation was performed utilising a 20 mm SS parallel plate geometry and a Peltier plate temperature system set to 25 °C on a DHR-2 stress-controlled rheometer which is also known as TA Instruments. Self-healing property was established through rheological thixotropic analysis.

FESEM images were collected by a Carl Zeiss SUPRA 55VP FESEM instrument. TEM images were conducted in an aberration corrected FEI Titan Themis operating at 300 kV. The chemical constituent elemental mapping was done using ZEISS EVO 18 microscope.

The FT-IR spectroscopic analysis was done by a JASCO FTIR 4700 spectrometer.

The current–voltage (I-V) measurements of our synthesized metallohydrogel material-based devices were performed at room temperature using a Keithley 2400 sourcemeter interfaced through Labview.

### Supplementary Information

Supplementary Figures.

## Data Availability

The datasets used and/or analysed during the current study available from the corresponding author on reasonable request.
