# Supplementary material for: Development of a novel self-healing Zn(II)-metallohydrogel with wide bandgap semiconducting properties for non-volatile memory device application
Source: Sci Rep. 2024 Jun 7;14:13109. doi: 10.1038/s41598-024-61870-1 (PMC11161586; doi:10.1038/s41598-024-61870-1)
Supplement: Supplementary file 1 — Supplementary Figures. [file 41598_2024_61870_MOESM1_ESM.docx]

Supporting Information

**Development of a Novel Self-Healing Zn(II)-Metallohydrogel with Wide Bandgap Semiconductor Properties for Non-Volatile Memory Device Application**

Arpita Roy,^§,ψ^ Subhendu Dhibar,*^,†,ψ^ Kripasindhu Karmakar,^†^ Subham Bhattacharjee,^¥^ Bidyut Saha*^,†^ and Soumya Jyoti Ray,*^,§^

^§^Department of Physics, Indian Institute of Technology Patna, Bihar-801106, India. *E-mail: ray@iitp.ac.in (S.Ray).

^†^Colloid Chemistry Laboratory, Department of Chemistry, The University of Burdwan, Golapbag, Burdwan-713104, West Bengal, India.

^¥^Department of Chemistry, Kazi Nazrul University, Asansol-713303, West Bengal, India

*E-mail: *sdhibar@scholar.buruniv.ac.in* (S Dhibar); *bsaha@chem.buruniv.ac.in* (B. Saha); *ray@iitp.ac.in* (S. J. Ray).

1. **Filament formation using TEM and EDAX analysis:**

We have measured IV characteristics for both devices with change in area between two electrodes. But there are no more differences in hysteresis loop for changing area between two electrodes. We can confirm that there is formation of conduction filament which arise due to Cu ion migration. Cu ion migration plays an important role for resistive switching mechanism. We already know that under an electric field, Cu ions can travel in the direction of the applied electric field. When we apply positive voltage, Cu^2+^ ions move towards the intermediate layer, where they are reduced to metallic Cu. Then, the conductivity of this layer will increase, and Cu ions accumulate towards the bottom electrode because they will act as conductive filaments to complete the SET process and switch from HRS to LRS. The device remains in the LRS state unless a sufficient voltage with opposite parity is applied to electrochemically dissolve the Cu filament for the RESET process. When negative voltage is applied, the device enters the HRS state and the conductivity of this device decreases simultaneously. Finally, Cu^2+^ ions drift back to the top electrode. We have also observed ion migration from TEM and EDAX analysis as shown in Figure S1(a)-(c). From TEM image, we observed the rod like structure after switching which confirms there is presence of filament during switching. From EDAX analysis, we can confirm absolutely that there is formation of Cu filament. There are 66 wt% Cu ions present in the sample after switching. In this way it provides a complete switching mechanism based on conductive filament model as shown in Figure S2.

1. Top of Form


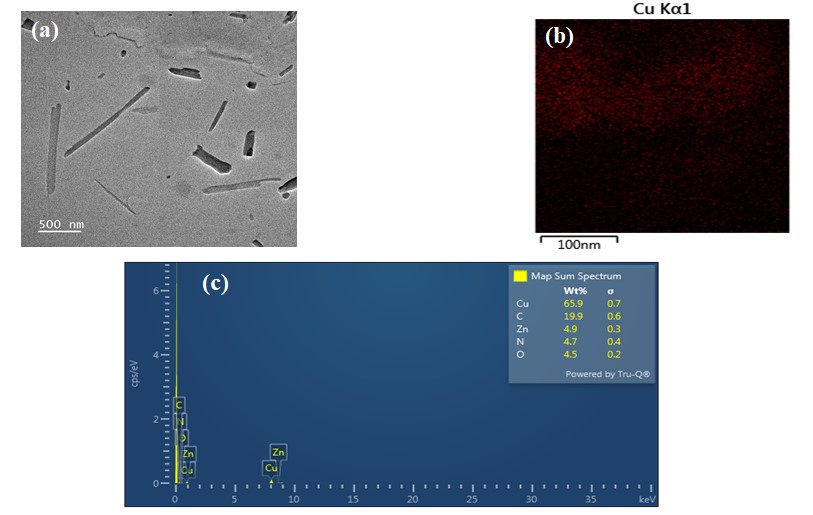
**Figure S1.** (a) TEM image of formation of Cu filament; (b-c): Elemental mapping of metallogel which confirms the presence of Cu filament of 65.9 wt%.

(b)


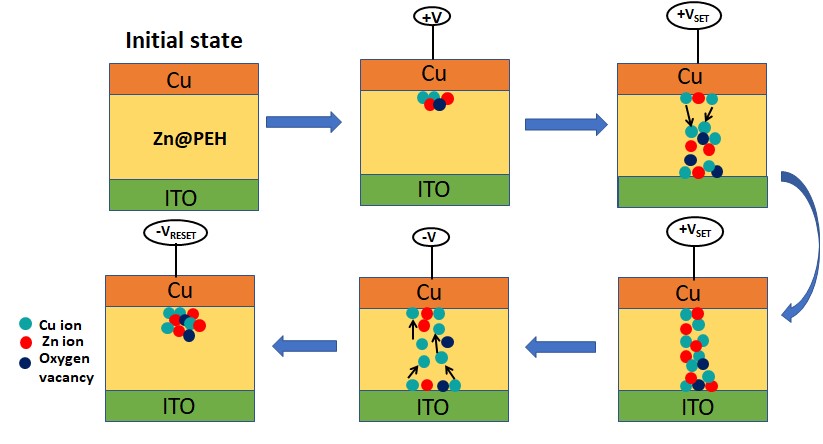


**Figure S2.** Conductive filament model of ITO/Zn@PEH/Cu-based device
